# Quercetin Attenuates Oxidative Stress and Immune Inflammation via Modulating Heme and ROS Pathways in Rats Fed Protein-Oxidized Soybean Meal

**DOI:** 10.3390/antiox15040504

**Published:** 2026-04-18

**Authors:** Zhiyong Wang, Peng Wang, Yanmin Zhou, Leli Wang, Su Zhuang

**Affiliations:** 1 College of Animal Science and Technology, Nanjing Agricultural University, Nanjing 210095, China; 2021205034@stu.njau.edu.cn (Z.W.); 2020205032@stu.njau.edu.cn (P.W.); zhouym@njau.edu.cn (Y.Z.); 2 Hunan Institute of Microbiology, Hunan Academy of Agricultural Sciences, Changsha 410009, China; 3 Institute of Subtropical Agriculture, Chinese Academy of Sciences, Changsha 410125, China; leliwang@isa.ac.cn

**Keywords:** bioactive compound, dietary protein oxidation, redox imbalance, inflammatory response, transcriptomics

## Abstract

Dietary protein oxidation impairs animal health, yet effective interventions remain limited. This study investigated whether quercetin (Q) supplementation protects against protein-oxidized soybean meal (OS)-induced oxidative stress and inflammatory injury in rats. A 2 × 2 factorial experiment was conducted with 48 three-week-old Sprague-Dawley rats randomly assigned to four dietary treatments (*n* = 12): fresh soybean meal (FS), FS + 400 mg/kg Q, OS, and OS + 400 mg/kg Q for 28 days. Serum biochemistry, intestinal and hepatic histology, antioxidant status, inflammatory markers, and transcriptomic pathways were analyzed. As a result, OS feeding elevated serum glucose and urea nitrogen, induced duodenal, jejunal and hepatic lesions, reduced total antioxidant capacity (T-AOC), glutathione peroxidase (GSH-Px) activity, glutathione (GSH) level, increased reactive oxygen species (ROS) and malondialdehyde (MDA) content (*p* < 0.05), and increased IgG and IL-6 levels (*p* < 0.05). Transcriptomic analysis revealed upregulation of heme biosynthesis and ROS synthesis pathways in jejunum and liver (*p* < 0.05). Q supplementation mitigated these adverse effects by improving antioxidant status, reducing inflammatory lesions, downregulating heme and ROS pathways, and normalizing the expression of key genes (*Ccl20*, *RT1-M2*) and protein (Ccl20) in jejunum (*p* < 0.05), and key genes (*Duox1*, *Cyp4a2*) and protein (Duox1) in liver (*p* < 0.05). These findings demonstrate that Q alleviates OS-induced oxidative stress, inflammation, and tissue damage through the modulation of heme and ROS pathways.

## 1. Introduction

Soybean meal is widely used in the diets of laying hens, broilers, swine, and aquatic species because of its balanced amino-acid profile. However, during processing and storage, the proteins of soybean meal inevitably undergo oxidative modification [1]. This oxidative modification alters the protein’s structure and properties, which is characterized by an increase in carbonyl content and a decrease in free sulfhydryl content [2], leading to a loss in nutritional value and the generation of potentially harmful substances [3]. The consumption of diets containing excessively oxidized proteins disrupts redox homeostasis and elicits systemic oxidative stress in animals [4,5]. Long-term intake of highly oxidized proteins leads to the systemic accumulation of reactive carbonyl species and provokes a sustained inflammatory response, severely compromising animal health and productivity [6]. Consequently, the impairment of antioxidant and anti-inflammatory functions in animals caused by protein-oxidized soybean meal (OS) warrants urgent investigation.

Quercetin (Q), a natural flavonol with well-documented in-vivo antioxidant and anti-inflammatory properties [7], is extensively used in animal feeds [8,9]. Mechanistically, Q directly scavenges excessive reactive oxygen species (ROS) and activates the nuclear factor erythroid 2-related factor 2 (Nrf2) signaling pathway, thereby upregulating downstream antioxidant enzymes including heme oxygenase-1 (HO-1), superoxide dismutase (SOD), and glutathione peroxidase (GSH-Px) to restore redox homeostasis [10]. Concurrently, Q modulates inflammatory responses by suppressing the nuclear factor-kappa B (NF-κB) signaling pathways, thereby reducing the production of pro-inflammatory cytokines such as TNF-α, IL-6, and IL-1β [11]. Accumulating evidence indicates that quercetin interrupts the vicious cycle between oxidative stress and inflammatory responses, protecting cells and tissues from damage. These properties highlight quercetin’s potential as a safe and effective agent for alleviating oxidative injury and excessive inflammation in disease intervention [12].

Accordingly, we hypothesized that dietary Q supplementation could alleviate oxidative damage and immune dysfunction induced by OS in rats. To verify this hypothesis, this study investigated the protective effects of quercetin against OS-induced impairment in antioxidant capacity and inflammatory status, and further explored the underlying molecular mechanisms by which quercetin counteracts OS-triggered oxidative and inflammatory injuries. Collectively, this study aims to alleviate oxidative and inflammatory damage induced by OS in animal production.

## 2. Materials and Methods

### 2.1. Tested Compounds

The fresh soybean meal (FS), sourced from Yihai Cereal & Oil Industry Co., Ltd. (Lianyungang, China), was stored at −20 °C from the date of production. Q (≥95% purity) was purchased from Hunan E.K. Herb Co., Ltd. (Changsha, China). The preparation method for OS was to place FS on a clean, sterilized 35 °C constant-temperature and -humidity chamber for 56 days. The humidity of the chamber was set at 60% to prevent moisture loss from the soybean meal. During storage, the soybean meal samples were turned over every 7 days, and the chamber was disinfected with an ultraviolet (UV) lamp for 30 min each time to prevent the soybean meal from mold growth. The characteristics of the FS and OS are given in Table 1.

### 2.2. Animals and Study Design

The study employed a 2 × 2 factorial arrangement with two factors: dietary protein source (FS vs. OS) and quercetin supplementation (0 vs. 400 mg/kg [13,14]). A total of 48 male Sprague-Dawley rats (SPF-grade, 3 weeks old, initial body weight 55 ± 5 g) were procured from Jiangsu Wukong Biotechnology Co., Ltd. (Nanjing, China) and randomly allocated into 4 treatment groups (*n* = 12 per group): the FS group was fed the basal diet; the FS + Q group was fed the basal diet supplemented with 400 mg/kg Q; the OS group was fed a basal diet in which OS replaced the FS; and the OS + Q group was fed a basal diet in which OS replaced FS and supplemented with 400 mg/kg Q. The composition and calculated nutrient levels of the basal diets are listed in Table 2. Before diet formulation, the dry matter of soybean meal in each group was adjusted to the same level.

### 2.3. Breeding Management

All the experimental procedures applied in this study were reviewed and approved by the Nanjing Agricultural University’s Animal Care and Use Committee (permit number: NJAU.No20230606094). Rats were maintained in SPF negative-pressure cages with free access to feed and drinking water. Environmental conditions were controlled as follows: 12 h light/12 h dark photoperiod, ambient temperature 20 ± 2 °C, and relative humidity 50~70%. The acclimation period lasted 1 week, followed by a formal experimental period of 4 weeks.

### 2.4. Sampling and Analysis

On the last day of the 5th week of the experiment, all rats were anesthetized with ether, followed by blood collection from the orbit and cervical dislocation for euthanasia. Collected blood was held at room temperature for 30 min, then spun at 3000× *g* (15 min, 4 °C) to isolate serum. The serum was aliquoted into centrifuge tubes and stored at −20°C for later analysis. Immediately after euthanasia, the rats were dissected to collect their liver, duodenum, jejunum, and ileum. Part of the liver and intestinal tissues was placed in 4% paraformaldehyde solution for hematoxylin and eosin (H&E) staining, while the remaining tissue was aliquoted into cryogenic tubes, rapidly frozen in liquid nitrogen for 6 h, and then stored at −20 °C for later analysis.

### 2.5. Serum Biochemical Indicators

Serum samples of equal weight from every three rats in the same group were pooled to form one biological replicate (*n* = 4 per group). The levels of glucose (GLU), total protein (TP), urea nitrogen (UN), triglycerides (TG), total serum cholesterol (TC), alanine aminotransferase (ALT), aspartate aminotransferase (AST), and albumin (ALB) in serum were measured using an automated biochemical analyzer (BX-4000, Himeliskang Co., Tokyo, Japan).

### 2.6. Intestinal and Hepatic Histopathology

Intestinal and hepatic tissues fixed in 4% paraformaldehyde were processed for paraffin embedding. Embedded blocks were sectioned at 5 µm, mounted on glass slides, and stained with H&E. The stained sections were photographed using a Nikon microscope (Eclipse E100, Nikon, Tokyo, Japan), and the histomorphological and pathological observations were performed using Scope Image 9.0 software (Bioimager Inc., Richmond Hill, ON, Canada). Each group includes 12 replicates.

### 2.7. Determination of Antioxidant Indicators

Tissue samples of equal weight from every three rats in the same group were pooled to form one biological replicate (*n* = 4 per group). All measurement steps were carried out according to the instructions provided by Nanjing Jiancheng Bioengineering Institute (Nanjing, China). Precisely weighed 0.1 g samples of rat jejunal and liver tissues were separately homogenized with cold physiological saline at an appropriate ratio. The homogenates of the jejunal and liver tissues were then centrifuged under the conditions described in the kit instructions. The total protein concentration in the supernatant was measured using a Total Protein (TP) Assay Kit (Cat. No.: A045-2-2). Subsequently, the following indicators were measured using the respective kits: Total Superoxide Dismutase (T-SOD) Assay Kit (Cat. No.: A001-1-2), Malondialdehyde (MDA) Assay Kit (Cat. No.: A003-1-2), Catalase (CAT) Assay Kit (Cat. No.: A007-1-1), Glutathione Peroxidase (GSH-Px) Assay Kit (Cat. No.: A005-1-2), Reactive Oxygen Species (ROS) Assay Kit (Cat. No.: E004-1-1), Total Antioxidant Capacity (T-AOC) Assay Kit (Cat. No.: A015-1-2), and Reduced Glutathione (GSH) Assay Kit (Cat. No.: A006-1-1).

### 2.8. Assessment of Immunological Parameters

Tissue samples of equal weight from every three rats in the same group were pooled to form one biological replicate (*n* = 4 per group). All measurement steps were carried out according to the instructions provided by Nanjing Hongsheng Biotechnology Co., Ltd. (Nanjing, China). Accurately weighed 0.1 g samples each of rat jejunal and liver tissues were homogenized with ice-cold physiological saline at an appropriate ratio, and then centrifuged to obtain the homogenates of the jejunal and liver tissues under the conditions described in the reagent kit instructions. The supernatant total protein concentration was then measured using the Total Protein (TP) Assay Kit (Cat. No.: A045-2-2) from Nanjing Jiancheng Bioengineering Institute. Finally, the parameters were measured using the following ELISA kits: rat immunoglobulin G (IgG) ELISA Kit (Cat. No.: CKE33921-1), rat immunoglobulin M (IgM) ELISA Kit (Cat. No.: CKE30629-1), rat interleukin 6 (IL-6) ELISA Kit (Cat. No.: CKE33628-1), rat interleukin 1β (IL-1β) ELISA Kit (Cat. No.: CKE30206-1), rat tumor necrosis factor α (TNF-α) ELISA Kit (Cat. No.: CKE33827-1), rat intercellular adhesion molecule 1 (ICAM-1; CD54) ELISA Kit (Cat. No.: CKE30356-1), and rat myeloperoxidase (MPO) ELISA Kit (Cat. No.: CKE31928-1).

### 2.9. Transcriptomics Analysis

Tissue samples of equal weight from every three rats in the same group were pooled to form one biological replicate (*n* = 4 per group). Transcriptomic analysis procedures for jejunal and liver tissues (0.1 g each) followed our published protocol [15]. Paired-end sequencing of final libraries was performed using the NovaSeq 6000 system (Illumina, San Diego, CA, USA) by Paiseno Biotech Co., Ltd. (Shanghai, China). Raw sequence reads were deposited in the NCBI Sequence Read Archive under BioProject accession PRJNA1121032.

### 2.10. Reverse Transcription Quantitative PCR (RT-qPCR)

Tissue samples of equal weight from every three rats in the same group were pooled to form one biological replicate (*n* = 4 per group). mRNA expression levels of differentially expressed genes screened by transcriptomics were measured using RT-qPCR. The selection criteria for RT-qPCR validation were as follow: top-ranked genes from KEGG pathway analysis (FDR < 0.05) were prioritized; genes with |log2FC| > 1.2 and adjusted *p* < 0.05 in the comparison groups were preferred; and genes with established roles in inflammation and oxidative stress were selected. Total RNA was extracted from jejunal and liver tissues (0.05 g each) using the TRIzol Reagent kit (Invitrogen, Carlsbad, CA, USA, Cat. No.: 15596-026) according to the kit instructions, and RNA concentration and purity were determined with a NanoDrop 2000 spectrophotometer (Nanodrop, Waltham, MA, USA), ensuring A260/A280 ratios between 1.8 and 2.0. Reverse transcription was carried out using the All-In-One 5X RT MasterMix kit (Nanjing Hongsheng Biotech Co., Ltd., Nanjing, China, Cat. No.: G592), followed by qPCR quantification of target genes using the BlasTaq 2X qPCR MasterMix kit (Nanjing Hongsheng Biotech Co., Ltd., Cat. No.: G892). Primer sequences for jejunal antioxidant or immune-inflammation-related genes *IRF7*, *Ccl20,* and *RT1-M2*, liver antioxidant or immune-inflammation-related genes *Duox1*, *Cyp4a2*, and *Tcf19*, as well as the reference gene *GAPDH*, are shown in Table 3. Primers were synthesized by Sangon Biotech Co., Ltd. (Shanghai, China). Gene expression in the liver and intestine of the FS group rats was set to 1, and the relative expression of each gene was calculated using the 2^−ΔΔCt^ method [16]. Each replicate included 2 technical repeats.

### 2.11. Western Blot (WB)

Tissue samples of equal weight from every four rats in the same group were pooled to form one biological replicate (*n* = 3 per group). WB technology was used to further measure the protein expression levels of differentially expressed genes screened from transcriptomics, based on RT-qPCR. Protein extraction was performed using T-PER Tissue Protein Extraction Reagent (Thermo Scientific, Waltham, MA, USA, Cat. No. 78510) following the manufacturer’s instructions to extract total protein from jejunal tissue (0.2 g) and liver tissue (0.2 g) samples, with the addition of Halt Protease and Phosphatase Inhibitor (100X) (Thermo Fisher, USA, Cat. No. 78440) during extraction to inhibit protease and phosphatase activity. Protein concentration was determined using the BCA Protein Assay Kit (Shanghai Beyotime, Shanghai, China, Cat. No. P0010) and a NanoDrop 2000 spectrophotometer (Thermo Fisher Scientific, USA). Electrophoresis was carried out using the Mini-PROTEAN electrophoresis system and Mini Trans-Blot transfer system (Bio-Rad, Hercules, CA, USA). Protein samples were separated by SDS-PAGE, with 8–12% separation gel and 5% stacking gel prepared, loading 60 μg of total protein per lane. Electrophoresis conditions were 60 V for pre-running and 80 V for separation for 2 h. After electrophoresis, proteins were transferred onto a PVDF membrane (Millipore, Burlington, MA, USA, Cat. No. IPVH00010), which was pre-soaked in methanol for 20 s and equilibrated in Tris-Glycine transfer buffer (containing 5% methanol) for 5 min. The transfer conditions were 100 V constant voltage and wet transfer for 2 h. After transfer, the PVDF membrane was blocked in T-TBS buffer (containing 3% skim milk) at room temperature for 1 h, and then washed with T-TBS for 5 min × 3 times. Primary antibodies for target proteins Ccl20 in jejunal tissue and Duox1 in liver tissue, as well as the internal reference protein GAPDH (specific information in Table 4), were diluted in T-TBS and incubated overnight at 4 °C. The following day, membranes were washed with T-TBS for 5 min × 4 times. Subsequently, Goat anti-rabbit IgG (H + L) secondary antibody (Thermo Fisher Scientific, USA, Cat. No. 31431) was diluted 1:5000 in T-TBS (containing 2% skim milk), incubated at room temperature for 1 h, and washed with T-TBS for 5 min × 5 times. Protein band signals were detected using SuperSignal Enhanced Chemiluminescence (ECL) substrate (Thermo Fisher Scientific, USA, Cat. No. 34075), and ECL DualVue Western Markers (Merck, Darmstadt, Germany, Cat. No. RPN810) were prepared according to the kit instructions. The membrane was incubated at room temperature for 1 min, and excess ECL reagent was removed, sealed with plastic wrap, and exposed to X-ray film (Hangzhou Huadong Medicine Co., Ltd., Hangzhou, China) for 5~10 min before development and fixation. The results were analyzed using Quantity One 4.6.8 software (Bio-Rad, USA) for grayscale analysis. Each replicate was measured 3 times. Relative expression of target proteins = [(target protein optical density)/(internal reference optical density)] × 10.

### 2.12. Data Analysis

Data were analyzed using IBM SPSS Statistics software (OEM version, 26.0, IBM Corp., Armonk, NY, USA) with a 2 × 2 two-way analysis of variance (ANOVA). Each measured parameter served as the dependent variable, while “soybean meal type (S),” “Q level (Q),” and their interaction (S × Q) were treated as fixed effects to evaluate the significance of main and interaction effects. Normality of data distribution was assessed using the Shapiro–Wilk test prior to parametric analyses. Non-normally distributed data were analyzed using non-parametric alternatives (Tamhane’s T2 test). When the interaction was significant, Tukey’s test was applied for pairwise comparisons. Results are presented as means ± standard error of the mean (SEM). Differentially expressed genes (DEGs) in transcriptomics were screened by DESeq analysis, with criteria of |fold change| > 2 and *p* < 0.05. Differential proteins detected by WB were analyzed using one-way ANOVA followed by Tukey’s test. *p* < 0.05 was considered significant.

## 3. Results

### 3.1. Serum Biochemical Indicators

As shown in Table 5, compared with the FS diet, the OS diet significantly increased GLU and UN levels (*p* < 0.05). A significant interaction effect of S × Q on GLB levels was observed (*p* < 0.05), indicating that the addition of Q had a significant mitigating effect on the abnormal elevation of GLB levels in the blood of rats fed the OS diet.

### 3.2. Intestinal and Hepatic Histopathology

**Figure 1:**
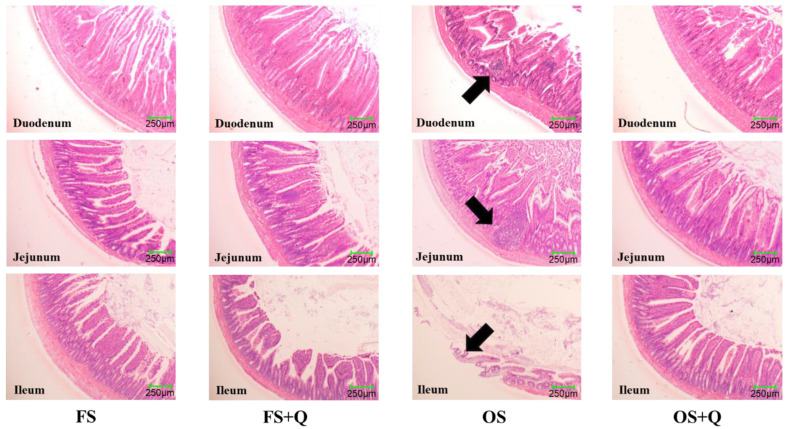
Effect of OS and Q on intestinal histopathology in rat. Arrows indicate pathological lesions.

Figure 1 shows histological sections of the duodenum, jejunum, and ileum of rats. Compared to the FS diet, rats fed the OS diet exhibited inflammatory lesions of varying sizes, with some areas of the ileal epithelium showing defects and a reduction in goblet cells. In contrast, compared with the diets not supplemented with Q, those groups supplemented with Q showed intact intestinal architecture and markedly reduced inflammation.

**Figure 2:**
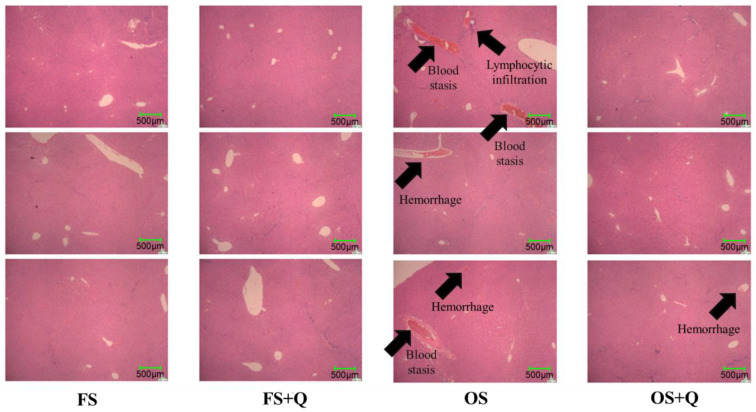
Effect of OS and Q on hepatic histopathology in rats. Arrows indicate pathological lesions.

Figure 2 shows histopathological sections of rat liver tissue. Compared to the FS diet, the rats fed the OS diet exhibited blood stasis, hemorrhage, and lymphocyte infiltration around the central vein. In contrast, compared with the diet without Q supplementation, those groups supplemented with Q showed normal liver tissue morphology, with reduced hemorrhage and inflammatory responses.

### 3.3. Antioxidant Indicators

Compared with the FS diet, OS diet impaired jejunal antioxidant capacity, evidenced by decreased T-AOC and GSH-Px activity, coupled with elevated ROS levels and compensatory CAT activation (*p* < 0.05, Table 6). Compared with no added Q, Q supplementation reversed these effects, reducing ROS and enhancing CAT activity (*p* < 0.05), with a significant S × Q interaction on T-AOC indicating Q’s protective effect against OS-induced antioxidant depletion.

Parallel to the jejunal findings, OS increased hepatic lipid peroxidation (MDA) while depleting GSH and GSH-Px (*p* < 0.05, Table 7). Q supplementation elevated T-AOC and reduced MDA (*p* < 0.05), demonstrating consistent cross-tissue antioxidant protection.

### 3.4. Immunological Parameters

Jejunal IgG and IL-6 levels were elevated (Table 8) and hepatic IgG increased but IL-1β decreased (Table 9) in rats fed the OS diet compared to the FS diet (*p* < 0.05). Notably, Q supplementation significantly reduced liver IgG (*p* < 0.05), with a significant S × Q interaction (*p* < 0.05), confirming that Q mitigated the OS-induced elevation of liver IgG.

### 3.5. Transcriptomics

**Figure 3:**
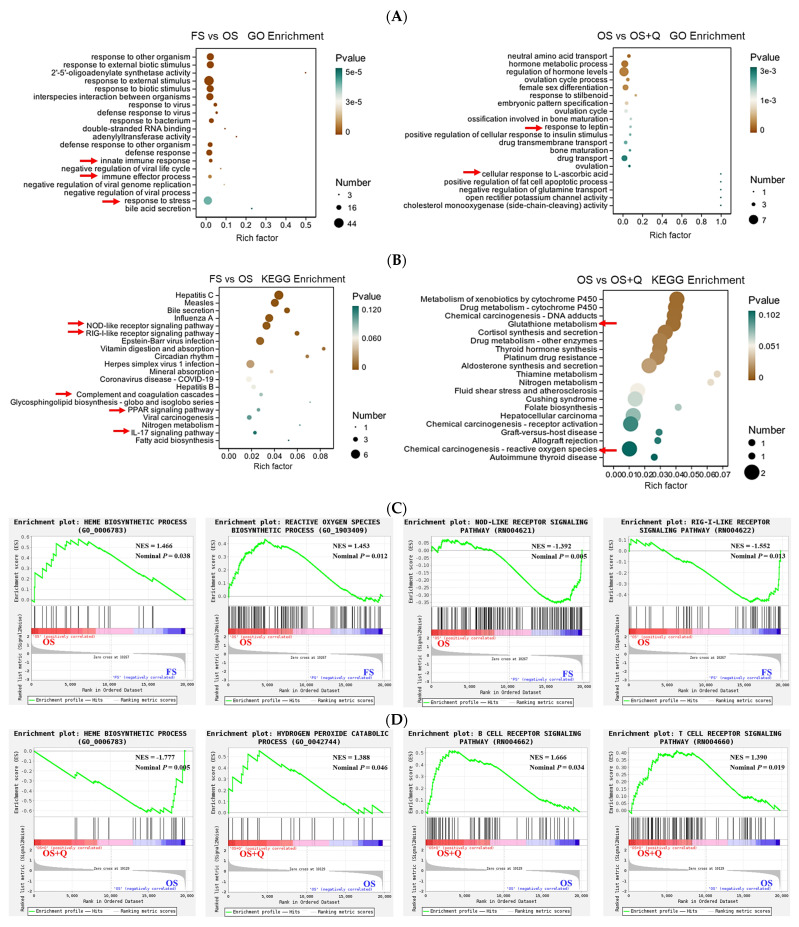
Jejunal transcriptome analysis. (**A**) GO enrichment of top 20 biological processes. (**B**) KEGG pathway enrichment of top 20 terms. Arrows indicate the top enriched GO terms associated with jejunal antioxidant or immune inflammation. (**C**,**D**) GSEA showing significantly enriched pathways; positive (NES > 1) and negative (NES < −1) enrichment indicated for (**C**) FS vs. OS and (**D**) OS vs. OS + Q comparisons (Nominal *p* < 0.05).

Gene Ontology (GO) and Kyoto Encyclopedia of Genomes and Genomes (KEGG) analyses of jejunal pathways revealed OS enrichment of innate immunity and stress responses (Figure 3A,B). Gene Set Enrichment Analysis (GSEA) pinpointed upregulation of the “heme biosynthetic process” and “ROS biosynthetic process” as hallmark OS responses (*p* < 0.05, Figure 3C). Q supplementation specifically downregulated heme biosynthesis while upregulating hydrogen peroxide catabolism and B-cell and T-cell receptor signaling pathways (*p* < 0.05, Figure 3D).

**Figure 4:**
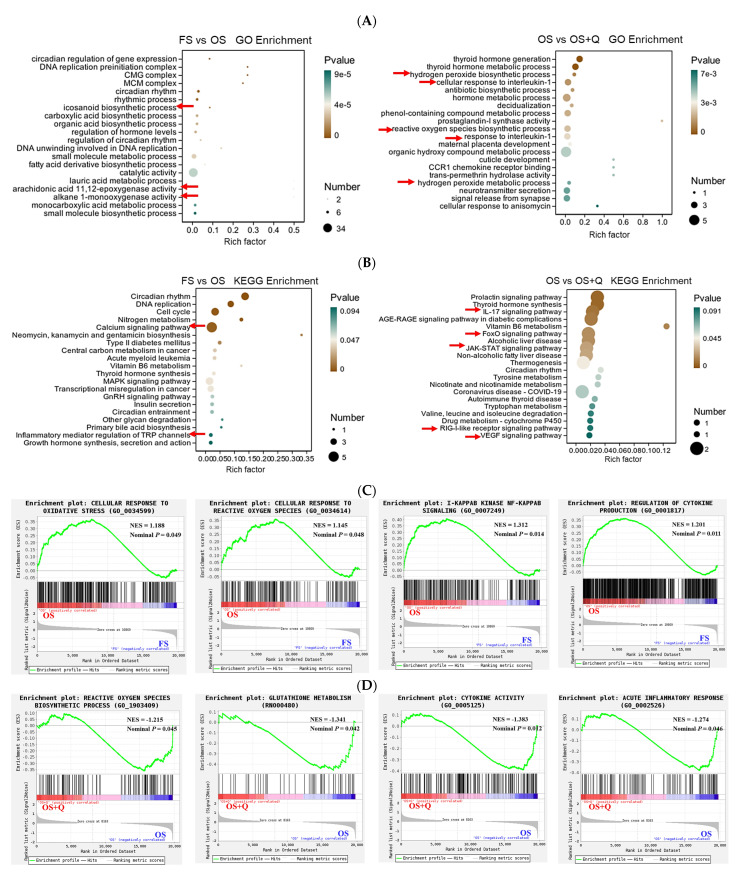
Hepatic transcriptome analysis. (**A**) GO enrichment of the top 20 biological processes. (**B**) KEGG pathway enrichment of the top 20 terms. Arrows indicate the top enriched GO terms associated with hepatic antioxidant or immune inflammation. (**C**,**D**) GSEA showing significantly enriched pathways; positive (NES > 1) and negative (NES < −1) enrichment indicated for (**C**) FS vs. OS and (**D**) OS vs. OS + Q comparisons (Nominal *p* < 0.05).

OS upregulated hepatic oxidative stress responses, I-κB kinase signaling, and cytokine production (*p* < 0.05, Figure 4C). Q counteracted these effects through downregulation of ROS biosynthesis, glutathione metabolism, cytokine activity, and acute inflammatory response pathways (*p* < 0.05, Figure 4D), corroborating the observed reduction in hepatic IgG.

### 3.6. RT-qPCR and WB

**Figure 5:**
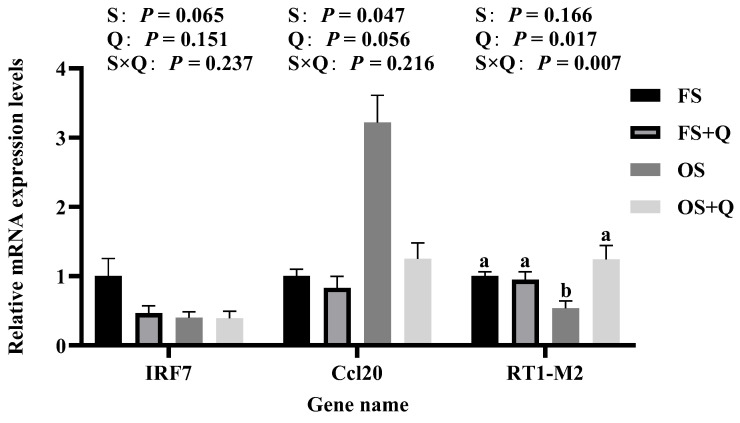
The mRNA expression levels of selected differential genes in rat jejunal tissue. ^a,b^ Values in a row with no common letters differ significantly (*p* < 0.05).

**Figure 6:**
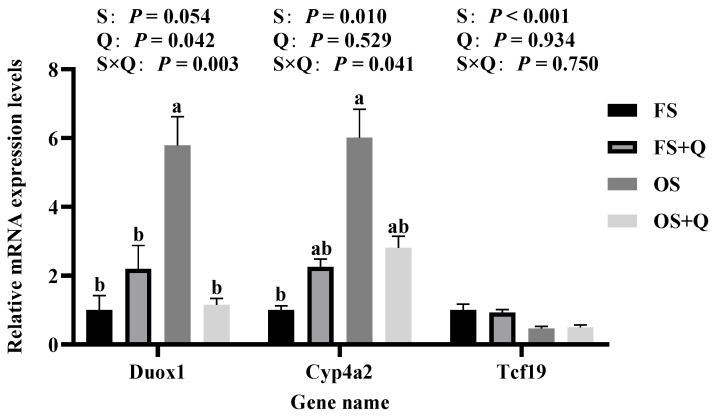
The mRNA expression levels of selected differential genes in rat liver tissue. ^a,b^ Values in a row with no common letters differ significantly (*p* < 0.05).

**Figure 7:**
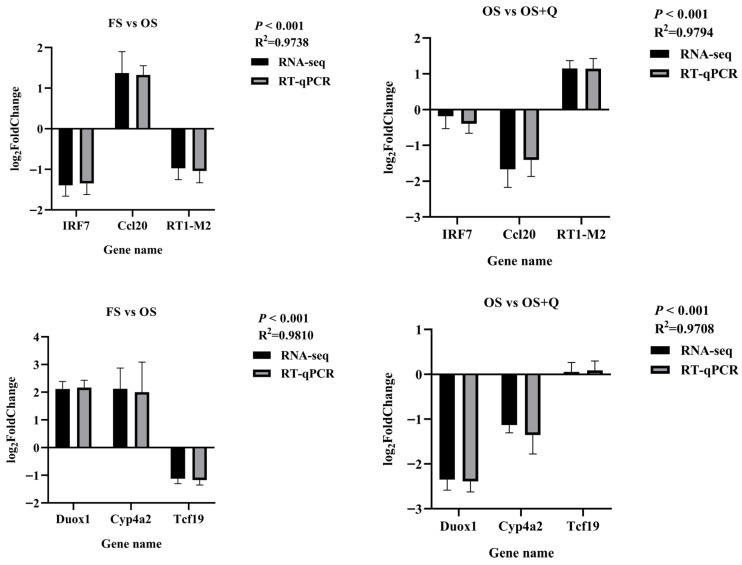
The results of the correlation analysis between transcriptome expression and RT-qPCR expression of mRNA of some differential genes.

**Figure 8:**
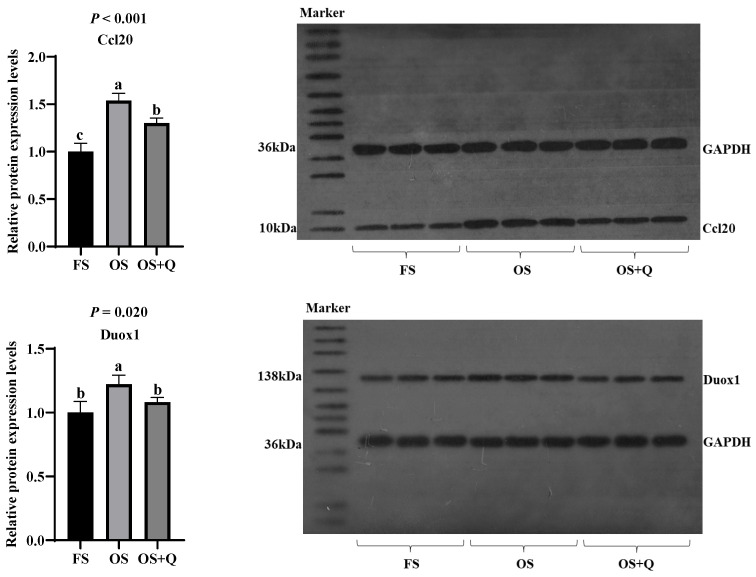
WB results of protein expression corresponding to some differential genes. ^a–c^ Values in a row with no common letters differ significantly (*p* < 0.05).

Transcriptomic findings were validated through RT-qPCR and WB. Differential expression of *Ccl20* and *RT1-M2* (jejunum, Figure 5) and *Duox1* and *Cyp4a2* (liver, Figure 6) showed a high concordance between RNA-seq and RT-qPCR (*R*^2^ > 0.97, Figure 7); significant S × Q interactions further validated the tissue-specific protective effects of Q. Protein expression confirmed Ccl20 and Duox1 as terminal effectors: OS upregulated both proteins, while Q normalized their expression (*p* < 0.05, Figure 8).

## 4. Discussion

### 4.1. Effects of Q on Blood Biochemical Indicators in Rats Fed OS

According to Table 1, compared with FS, the content of protein carbonyl in OS was significantly increased, while the content of protein free sulfhydryl was significantly decreased. Consequently, the antioxidant status of rats fed an OS diet is inevitably affected. While we did not assess quercetin bioavailability, the selected dose (400 mg/kg) aligns with previous similar studies in rats [13,14] and livestock [17]. Further research should focus on validating quercetin bioavailability and exploring dose-dependent effects to establish its optimal therapeutic range.

Blood biochemical indicators can partially reflect the status of oxidative stress in animals [18]. Research has shown that when animals experience oxidative stress, their glucose metabolism accelerates, insulin resistance increases, and the balance of glucose synthesis and degradation is disrupted, leading to elevated blood GLU levels [19]. The results of this experiment showed that, compared with the FS diet, the OS diet significantly increased serum GLU levels in rats, which aligns with the conclusions of the aforementioned studies. Similarly, compared with the FS diet, the OS diet significantly increased serum UN levels in rats, which was consistent with previous experimental results finding that heat-induced OS diet remarkably raised serum UN levels in broilers [4]. This phenomenon suggests that OS affects the synthesis and degradation metabolism of amino acids and proteins in rats [15], and may have adverse effects on their liver and renal function by increasing the nitrogen metabolic load. In addition, OS and Q showed a significant interaction on GLB levels, which indicates that Q can significantly reverse the OS-induced elevation of immunoglobulin levels in rats. This is related to Q’s ability to suppress inflammatory responses and restore humoral immunity and antibody levels in animals to normal [20].

### 4.2. Effects of Q on Tissue Pathological Changes in Rats Fed OS

The accumulation of chronic oxidative stress precipitated significant structural damage to the intestinal mucosa, as evidenced by villus shortening and inflammatory cell infiltration, ultimately compromising gut barrier integrity [21]. Histopathological sections of intestinal tissue showed that, compared with the FS group, the OS group exhibited inflammatory lesions of varying sizes in the intestines, with some areas of ileal epithelium showing defects, reduced goblet cells, and other pathological changes. This is consistent with previous studies reporting that OS induced intestinal inflammation and epithelial damage in bluntnose bream [5], indicating that feeding OS led to substantial organ damage in rats. However, compared with the OS group, the intestinal tissue structure of the OS + Q group was intact, and the inflammatory lesions caused by oxidative stress were effectively alleviated. This is related to Q’s ability to inhibit oxidative damage, reduce inflammatory responses, and protect intestinal health [7,22]. These results indicate that adding Q has a significant mitigating effect on organ lesions in the intestines of rats induced by OS diet-related oxidative stress and enteritis.

Histopathological sections of liver tissue showed that, compared with the FS group, the OS group exhibited hepatic blood stasis, hemorrhage, and lymphocyte infiltration, indicating that OS caused oxidative stress damage and inflammatory responses in rat liver [23]. This result is similar to previous studies showing the negative effects of photo-oxidized milk protein on mouse liver histopathology [24], suggesting that oxidized protein feed not only causes intestinal damage but also harms liver health. However, compared with the OS group, the liver tissue of the OS + Q group appeared normal, and hemorrhage and inflammation were reduced. This is in line with Q’s antioxidant, anti-inflammatory, and hepatoprotective effects [25]. These results suggest that Q significantly alleviates oxidative stress damage and inflammatory responses in liver tissues caused by the OS diet in rats. While these results are compelling, the relatively short feeding period (4 weeks) precludes conclusions about long-term adaptation or chronic toxicity; long-term validation is warranted.

### 4.3. Effects of Q on Oxidative Stress and Immune Inflammation in the Jejunal and Liver Tissues of Rats Fed OS

Oxidative stress reflects an imbalance between ROS production and antioxidant defense. In the jejunum, OS significantly reduced T-AOC and GSH-Px while increasing ROS levels, confirming OS-induced oxidative stress, as previously reported in broilers [4]. Q supplementation reduced ROS and elevated CAT activity, consistent with Q’s ROS-scavenging properties [26] and its ability to increase cardiac CAT in LPS-challenged rats [27]. The significant OS × Q interaction on T-AOC indicates that Q reversed the OS-induced decline in T-AOC, similar to earlier findings [8]. A critical observation is that while CAT activity increased, other antioxidant indicators (e.g., SOD, GSH-Px) were not uniformly assessed; thus, the specificity of Q’s effect on CAT warrants further investigation.

The results of this study indicate that in the liver, compared to the FS diet, the OS diet significantly increased MDA content and significantly decreased GSH and GSH-Px activity. This suggests that oxidative stress occurred in the rat liver, consistent with previous results from feeding OS to broilers [4]. MDA is a product of lipid peroxidation and an important indicator of oxidative stress, which can cause liver cell damage [28]. GSH and GSH-Px are important antioxidant molecules representing the liver’s reserve antioxidant capacity [29]; their decreased activity indicates severe impairment of the liver’s antioxidant capacity in rats. However, compared to not adding Q, supplementation with Q significantly increased T-AOC levels and decreased MDA content. This result is similar to previous findings that Q can increase T-AOC levels in rat liver tissue [8] and is also comparable to its role in alleviating oxidative stress-induced renal tubular epithelial cell damage in hyperoxaluric model rats by reducing MDA levels [26]. Overall, these results indicate that Q has an effect on alleviating oxidative stress in the liver of rats fed an OS diet.

Research shows that persistent oxidative stress can activate the immune system, promoting the production of antibodies and cytokines [30]. Oxidative stress can also damage intestinal barrier function, leading to increased intestinal mucosal permeability, which allows pathogens and antigens to more easily penetrate the intestinal mucosa, thereby triggering immune responses and inflammation [31]. The results of this study indicate that in the jejunum, compared to the FS diet, the OS diet significantly increased IgG and IL-6 levels. This abnormal increase in IgG suggests that OS induces chronic inflammatory responses in the rat intestine [32]. The abnormal elevation of IL-6 levels suggests that OS triggers the expression of pro-inflammatory cytokines, a phenomenon similar to earlier studies showing that feeding laying hens stored soybean meal led to increased interleukin-4 (IL-4) mRNA expression in the jejunum [33]. In general, the OS diet specifically elevated IgG and IL-6 without significant changes in IgM, IL-1β, or TNF-α, suggesting predominant B-cell and Th17-mediated chronic responses rather than acute macrophage-driven inflammation characterized by TNF-α and IL-1β dominance [34,35].

Regarding immune inflammation in rat liver, the results of this study show that compared to the FS diet, the OS diet significantly increased liver IgG content and significantly reduced IL-1β content. IgG in liver tissue mainly originates from two sources: first, it is directly secreted by plasma cells infiltrating the liver [36]; second, it is transported from the mesenteric circulation. In this study, simultaneous increases in IgG in both the intestine and the liver were observed, suggesting the possible abnormal activation of an “intestinal-liver axis immune dialogue” [37]. However, this hypothesis still needs to be confirmed by subsequent measurements of portal circulation IgG or lymphocyte tracking. The reduction in IL-1β—a key host defense cytokine—suggests a protective negative feedback immune response [38]; however, the specific causes of this phenomenon still require further research. Overall, Q significantly reduced hepatic IgG, and the OS × Q interaction indicates reversal of OS-induced IgG elevation, consistent with Q’s anti-inflammatory effects [39].

### 4.4. Effects of Q on the Transcription of Oxidative Stress and Immune Inflammation-Related Genes in Rats Fed OS

As the largest digestive organ in animals, the intestine is constantly exposed to pro-oxidant stimuli. Heme-derived iron overload promotes the generation of ROS via the Fenton reaction, which further induces mitochondrial dysfunction and the ferroptotic cascade. This heme–ROS–ferroptosis axis serves as a core molecular pathway that mediates the transition from metabolic stress to the activation of innate immune responses, thereby participating in the pathogenesis of multiple tissue injuries and inflammatory diseases [40]. Heme can trigger programmed necrosis in macrophages by promoting autocrine TNF signaling and ROS generation, thereby linking heme metabolism, oxidative stress, and innate immune responses during inflammatory processes [41]. Our GSEA results showed that OS upregulated jejunal heme biosynthesis and ROS biosynthesis, while downregulating NOD-like and RIG-I-like receptor signaling pathways. This may be attributed to the activation of innate self-protective anti-inflammatory responses in the intestine under oxidative stress, suggesting that OS induces intestinal stress via ROS production, and the long-term accumulation of ROS further triggers innate negative feedback inhibition in the intestine [42]. The underlying mechanisms warrant further investigation in future studies. Q supplementation downregulated heme biosynthesis and upregulated hydrogen peroxide catabolism, B-cell and T-cell receptor signaling pathways, consistent with the observed reduction in ROS and normalization of antibody/cytokine levels.

In the liver, OS similarly upregulated cellular responses to oxidative stress and ROS, NF-κB signaling, and cytokine production pathways, unlike the jejunum, however, where NOD-like/RIG-I pathways were downregulated. This tissue-specific difference may reflect the distinct roles of ROS in different organs [43], and could also be an artifact of baseline expression differences or pathway crosstalk—points that require mechanistic dissection using tissue-specific knockouts. Furthermore, Q downregulated ROS biosynthesis, glutathione metabolism, cytokine activity, and acute inflammatory response pathways in the liver, indicating alleviation of oxidative stress and inflammation [44]. These transcriptomic results align well with the biochemical and histopathological data.

RT-qPCR and WB analyses confirmed that Q reversed OS-induced downregulation of jejunal *RT1-M2* (an MHC class I-related gene) and *Ccl20* (a pro-inflammatory chemokine), and reversed OS-induced upregulation of hepatic *Duox1* (NADPH oxidase) and *Cyp4a2* (cytochrome P450). Furthermore, Q also normalized OS-induced elevation of jejunal Ccl20 and hepatic Duox1 protein. Notably, Duox1 is a core component of the heme-dependent ROS biosynthetic pathway, whereas Ccl20 acts as a downstream inflammatory chemokine induced by heme–ROS signaling. These convergent results across mRNA and protein levels strengthen the reliability of the findings.

### 4.5. Overall Limitations and Future Directions

Several limitations should be acknowledged. First, the use of a single quercetin dose (400 mg/kg) precludes dose-response analysis and the identification of an optimal therapeutic window. Second, the study employed a healthy young rat model; the results may not generalize to aged, diabetic, or immune-compromised populations. Third, while we observed correlations between transcriptomic, biochemical, and histopathological changes, no functional experiments (e.g., pathway inhibition, gene knockdown, or rescue) were conducted to establish causality. Fourth, the relatively short intervention period limits conclusions about long-term efficacy and safety. Fifth, while our protein validation focused on inflammatory and oxidative effectors (Ccl20, Duox1), future studies should incorporate heme metabolism enzymes (Alas1, Ho-1) to establish direct mechanistic connections between transcriptomic pathway enrichment and functional protein changes. Sixth, regarding the group selection for WB analysis, our initial design focused on FS versus OS (to assess OS-induced changes) and OS versus OS + Q (to evaluate Q treatment efficacy), following the same comparison strategy used in the transcriptomic analysis, thereby omitting the FS + Q group. We acknowledge that this approach failed to fully account for potential 2 × 2 factorial interaction effects, and the absence of the FS + Q group limits the interpretation of whether these changes reflect a specific rescue effect under OS conditions or a broader effect of quercetin.

## 5. Conclusions

This study shows that OS diet induces oxidative stress and pro-inflammation in rat jejunum and liver. Q supplementation enhances antioxidant capacity, reduces ROS, modulates jejunal heme and immune pathways, and restores hepatic glutathione metabolism while suppressing inflammation. These findings suggest Q as a potential dietary supplement that can attenuate OS-induced oxidative stress and immune inflammation.

## Figures and Tables

**Table 1:** Characteristics of the FS and OS.

Items	FS	OS
Crude protein content (%)	43.13	43.14
Fat content (%)	0.88	0.88
Moisture (%)	12.99	12.96
Protein carbonyl (nmol/mg of protein)	7.09	10.32
Free sulfhydryl (nmol/mg of protein)	12.38	5.56
Mold situation	Not moldy	Not moldy
Aflatoxin B1 content (μg/kg)	≤30	≤30

**Table 2:** Composition and calculated nutrient content of experimental diets (%, as-fed basis).

Items	Content (%)	
	FS Diet	OS Diet
Corn	37.58	37.58
FS	29.91	–
OS	–	29.91
Subflour	1.99	1.99
Wheat	19.94	19.94
Wheat bran	1.00	1.00
Soybean oil	1.99	1.99
Limestone	1.20	1.20
Dicalcium phosphate	1.50	1.50
Sodium chloride	0.25	0.25
Magnesium oxide	0.15	0.15
Choline chloride	0.20	0.20
Cr_2_O_3_	0.30	0.30
Premix ^1^	3.99	3.99
Calculated nutrient levels		
Metabolizable energy (MJ/kg)	13.38	13.38
Crude protein	20.94	20.94
Ether extract	4.49	4.49
Crude fiber	3.99	3.99
Crude ash	5.78	5.78
Calcium	1.10	1.10
Total phosphorus	0.75	0.75
Lysine	1.30	1.30
Methionine + cystine	0.86	0.86

^1^ The premix was supplied by Jiangsu Xietong Pharmaceutical Bio-engineering Co., Ltd. (Nanjing, China), with the following composition per kilogram: valine, 40.90 g; leucine, 30.98 g; isoleucine, 49.52 g; phenylalanine, 0.80 g; methionine, 59.85 g; threonine, 42.31 g; tyrosine, 0.60 g; cystine, 0.50 g; lysine, 101.45 g; arginine, 1.10 g; histidine, 0.50 g; tryptophan, 20.15 g; vitamin D, 78,932.72 IU; vitamin E, 2582.60 IU; vitamin A, 542,633.19 IU; vitamin K, 215.94 mg; carotene, 0.37 mg; choline, 146.56 mg; nicotinic acid, 2308.88 mg; pyridoxine hydrochloride, 481.10 mg; riboflavin, 466.32 mg; biotin, 8.12 mg; thiamine, 1035.91 mg; cyanocobalamin, 1.01 mg; pantothenic acid, 929.74 mg; linoleic acid, 0.02 g; folic acid, 251.29 mg; Cl, 29.50 g; Na, 20.50 g; K, 0.70 g; Mg, 0.50 g; Fe, 2496.29 mg; Zn, 1496.90 mg; Mn, 1097.03 mg; Cu, 180.23 mg; I, 16.75 mg; Se, 5.10 mg.

**Table 3:** Primer sequences for PCR.

Gene Name	Gene Bank ID	Primer	Sequences
*GAPDH*	NM_017008.4	F	GACATGCCGCCTGGAGAAAC
		R	AGCCCAGGATGCCCTTTAGT
*IRF7*	NM_001033691.1	F	GGACGCTGGATCAACACCTGTG
		R	ACGGGCAGTCTGGGAGAAAGTAG
*Ccl20*	NM_019233.2	F	TTCACAACACAGATGGCCGA
		R	GGTTCTTAGGCTGAGGAGGTG
*RT1-M2*	NM_001001717.2	F	GGCCCTGACCCAGTCCTTT
		R	CTGCGCGCAGTAGAGTCTC
*Duox1*	NM_153739.3	F	AACCCTACCTGCCTAACCC
		R	CTGTCCAGTGCTGCGGTC
*Cyp4a2*	NM_001044770.2	F	ACCAGATTCTCCTCGCCATAGCC
		R	GCTTCTTGAGACGCAGGTGGATC
*Tcf19*	NM_213561.3	F	ATCACGGTCCCTCGGTCCAAG
		R	TCTCATCATCCAGTTCTGCCAACAC

**Table 4:** Primary antibody information.

Primary Antibody Name	Brand	Catalog Number	Dilution	Molecular Weight (kDa)
Ccl20	ABCAM	# Ab9829	1:1000	10
Duox1	Proteintech	67226-1-AP	1:600	138
GAPDH	ABCAM	# Ab181602	1:2000	36

**Table 5:** Effects of OS and Q on serum biochemical indicators in rats.

Items	GLU (mmol/L)	TP (g/L)	ALB (g/L)	GLB (g/L)	GPT (U/L)	GOT (U/L)	UN (mmol/L)	TC (mmol/L)
FS	2.23	68.28	44.13	24.15 ^xy^	70.18	338.70	5.11	2.64
FS + Q	2.64	69.50	43.10	26.40 ^xy^	67.60	289.05	5.21	2.39
OS	3.62	72.45	40.95	31.50 ^x^	63.00	269.55	6.35	2.24
OS + Q	3.44	65.10	44.45	20.65 ^y^	60.80	190.40	5.82	2.03
SEM	0.18	1.22	0.83	1.43	2.52	33.32	0.15	0.24
Main effects								
Soybean meal type (S)								
FS	2.44 ^b^	68.89	43.62	25.28	68.89	313.88	5.16 ^b^	2.52
OS	3.53 ^a^	68.78	42.70	26.08	61.90	229.98	6.09 ^a^	2.14
Q level (Q)								
−	2.93	70.37	42.54	27.83	66.59	304.13	5.73	2.44
+	3.04	67.30	43.78	23.53	64.20	239.73	5.52	2.21
*p* value								
S	<0.001	0.961	0.597	0.728	0.206	0.240	<0.001	0.487
Q	0.617	0.203	0.476	0.080	0.656	0.361	0.252	0.666
S × Q	0.215	0.084	0.204	0.013	0.972	0.832	0.100	0.965
Effect size								
S	0.661	<0.001	0.024	0.010	0.130	0.113	0.694	0.041
Q	0.022	0.131	0.043	0.234	0.017	0.070	0.108	0.016
S × Q	0.125	0.228	0.131	0.415	<0.001	0.004	0.209	<0.001

^a,b,x,y^ Within a column, means without a common superscript letter differ significantly (*p* < 0.05).

**Table 6:** Effects of OS and Q on jejunal antioxidant indicators in rats.

Items	T-AOC (U/mg Prot.)	MDA (nmol/mg Prot.)	ROS (a.u./mg Prot.)	T-SOD (U/mg Prot.)	CAT (U/mg Prot.)	GSH (μmol/g Prot.)	GSH-Px (U/mg Prot.)
FS	4.91 ^x^	3.76	185.91	28.13	1.19	8.40	85.50
FS + Q	4.68 ^x^	3.69	179.88	29.23	1.52	8.91	89.93
OS	3.69 ^y^	4.47	210.23	29.63	1.28	7.83	67.61
OS + Q	4.45 ^xy^	3.51	186.45	28.04	2.11	8.11	74.43
SEM	0.16	0.18	4.37	0.52	0.12	0.20	3.51
Main effects							
Soybean meal type (S)							
FS	4.80 ^a^	3.73	182.90 ^b^	28.68	1.36 ^b^	8.66	87.72 ^a^
OS	4.07 ^b^	3.99	198.34 ^a^	28.84	1.70 ^a^	7.97	71.02 ^b^
Q level (Q)							
−	4.30	4.12	198.07 ^A^	28.88	1.24 ^B^	8.12	76.56
+	4.57	3.60	183.17 ^B^	28.64	1.82 ^A^	8.51	82.18
*p* value							
S	0.007	0.463	0.036	0.893	0.034	0.115	0.014
Q	0.233	0.172	0.041	0.832	0.002	0.338	0.326
S × Q	0.040	0.239	0.186	0.265	0.098	0.770	0.829
Effect size							
S	0.614	0.069	0.442	0.442	0.270	0.281	0.547
Q	0.172	0.219	0.424	0.424	0.581	0.115	0.121
S × Q	0.430	0.168	0.207	0.207	0.137	0.011	0.006

^a,b,A,B,x,y^ Within a column, means without a common superscript letter differ significantly (*p* < 0.05).

**Table 7:** Effects of OS and Q on liver antioxidant indicators in rats.

Items	T-AOC (U/mg Prot.)	MDA (nmol/mg Prot.)	ROS (a.u./mg Prot.)	T-SOD (U/mg Prot.)	CAT (U/mg Prot.)	GSH (μmol/g Prot.)	GSH-Px (U/mg Prot.)
FS	1.52	4.08	159.69	20.84	7.69	15.44	424.51
FS + Q	1.65	3.57	158.80	23.88	8.61	15.54	429.68
OS	1.26	5.54	169.82	21.79	8.08	13.55	364.93
OS + Q	1.64	4.26	159.81	21.98	8.24	15.07	399.46
SEM	0.06	0.25	3.70	0.55	0.24	0.31	10.27
Main effects							
Soybean meal type (S)							
FS	1.59	3.83 ^b^	159.25	22.36	8.15	15.49 ^a^	427.10 ^a^
OS	1.45	4.90 ^a^	164.82	21.89	8.16	14.31 ^b^	382.20 ^b^
Q level (Q)							
−	1.39 ^B^	4.81 ^A^	164.76	21.32	7.89	14.50	394.72
+	1.65 ^A^	3.92 ^B^	159.31	22.93	8.43	15.31	414.57
*p* value							
S	0.204	0.003	0.509	0.654	0.987	0.036	0.022
Q	0.032	0.009	0.518	0.157	0.324	0.123	0.246
S × Q	0.224	0.182	0.588	0.205	0.478	0.171	0.382
Effect size							
S	0.193	0.676	0.056	0.026	<0.001	0.441	0.500
Q	0.458	0.594	0.054	0.234	0.121	0.271	0.164
S × Q	0.179	0.211	0.038	0.192	0.065	0.220	0.097

^a,b,A,B^ Within a column, means without a common superscript letter differ significantly (*p* < 0.05).

**Table 8:** Effects of OS and Q on jejunal immune inflammatory indicators in rats.

Items	IgG (mg/mg Prot.)	IgM (μg/mg Prot.)	IL-6 (pg/mg Prot.)	IL-1β (pg/mg Prot.)	TNF-α (pg/mg Prot.)	ICAM (ng/mg Prot.)	MPO (ng/mg Prot.)
FS	0.28	38.62	2.22	0.95	6.72	1.87	5.78
FS + Q	0.26	39.82	2.11	1.18	5.93	1.88	5.57
OS	0.43	47.48	3.34	1.02	6.38	1.90	5.97
OS + Q	0.43	48.42	3.01	0.98	6.24	1.90	5.79
SEM	0.03	2.79	0.19	0.06	0.27	0.11	0.21
Main effects							
Soybean meal type (S)							
FS	0.27 ^b^	39.22	2.17 ^b^	1.07	6.33	1.88	5.68
OS	0.43 ^a^	47.95	3.18 ^a^	1.00	6.31	1.90	5.88
Q level (Q)							
−	0.36	43.05	2.78	0.99	6.55	1.89	5.88
+	0.35	44.12	2.56	1.08	6.09	1.89	5.68
*p* value							
S	<0.001	0.168	0.003	0.626	0.985	0.928	0.668
Q	0.680	0.858	0.385	0.455	0.457	0.993	0.693
S × Q	0.629	0.983	0.646	0.298	0.597	0.999	0.974
Effect size							
S	0.879	0.224	0.812	0.031	<0.001	0.001	0.024
Q	0.022	0.004	0.165	0.072	0.071	<0.001	0.020
S × Q	0.031	<0.001	0.046	0.134	0.037	<0.001	<0.001

^a,b^ Within a column, means without a common superscript letter differ significantly (*p* < 0.05).

**Table 9:** Effects of OS and Q on liver immune inflammatory indicators in rats.

Items	IgG (mg/mg Prot.)	IgM (μg/mg Prot.)	IL-6 (pg/mg Prot.)	IL-1β (pg/mg Prot.)	TNF-α (pg/mg Prot.)	ICAM (ng/mg Prot.)	MPO (ng/mg Prot.)
FS	0.19 ^y^	23.76	1.74	0.60	6.34	0.84	3.81
FS + Q	0.19 ^y^	21.57	1.74	0.47	4.97	0.82	4.07
OS	0.27 ^x^	24.65	1.86	0.43	4.44	0.72	4.12
OS + Q	0.20 ^y^	23.09	1.64	0.39	4.51	0.78	3.81
SEM	0.01	1.06	0.10	0.03	0.36	0.03	0.12
Main effects							
Soybean meal type (S)							
FS	0.19 ^b^	22.67	1.74	0.54 ^a^	5.66	0.83	3.94
OS	0.24 ^a^	23.87	1.75	0.41 ^b^	4.48	0.75	3.97
Q level (Q)							
−	0.23 ^A^	24.21	1.80	0.52	5.39	0.78	3.97
+	0.20 ^B^	22.33	1.69	0.43	4.74	0.80	3.94
*p* value							
S	0.002	0.625	0.958	0.046	0.109	0.267	0.931
Q	0.006	0.450	0.633	0.170	0.347	0.775	0.930
S × Q	0.006	0.897	0.642	0.476	0.302	0.542	0.328
Effect size							
S	0.722	0.031	<0.001	0.361	0.289	0.151	0.001
Q	0.631	0.073	0.030	0.222	0.111	0.011	0.001
S × Q	0.632	0.002	0.027	0.065	0.132	0.048	0.119

^a,b,A,B,x,y^ Within a column, means without a common superscript letter differ significantly (*p* < 0.05).

## Data Availability

The original contributions presented in this study are included in the article. Further inquiries can be directed to the corresponding author.
